# Motion score for spectral quality control of optoacoustic-ultrasound data

**DOI:** 10.1038/s41598-025-33753-6

**Published:** 2026-01-10

**Authors:** Jan Kukačka, Maximilian Bader, Dominik Jüstel, Vasilis Ntziachristos

**Affiliations:** 1 Chair of Biological Imaging, Central Institute for Translational Cancer Research (TranslaTUM), School of Medicine and Health & School of Computation, Information and Technology, Technical University of Munich, Munich, Germany; 2 Institute of Biological and Medical Imaging, Bioengineering Center, Helmholtz Zentrum München, Neuherberg, Germany; 3 Institute of Computational Biology, Computational Health Center, Helmholtz Zentrum München, Neuherberg, Germany; 4 Institute of Electronic Structure and Laser (IESL), Foundation for Research and Technology Hellas (FORTH), Heraklion, Greece; 5 Munich Institute of Biomedical Engineering (MIBE), Technical University of Munich, Garching b. München, Germany; 6 Munich Institute of Robotics and Machine Intelligence (MIRMI), Technical University of Munich, Munich, Germany; 7 DZHK (German Centre for Cardiovascular Research), Partner site Munich Heart Alliance, Munich, Germany; 8 Chair of Biological Imaging, Technical University of Munich, Ismaninger Straße 22, D-81675 Munich, Germany

**Keywords:** Optoacoustic imaging, Motion quantification, Clinical imaging, Automatized image processing, Standardization, Photoacoustic tomography, Photoacoustics, Medical imaging

## Abstract

Motion can markedly affect the image quality acquired by handheld multispectral optoacoustic-ultrasound (MS-OPUS) hybrid systems. Since images at different wavelengths are acquired sequentially, tissue and operator movement can affect the spectra assigned to each volume element (voxels) in the images. To improve the accuracy of MS-OPUS spectral measurements, we sought to develop a method that could quantify and mitigate motion effects. We introduce *Motion score*, a post-processing algorithm for motion quantification and automated frame selection in MS-OPUS. Our method estimates motion in frames by tracking the similarity of co-registered ultrasound images and automatically selects low-motion frames. Algorithmic performance was validated on a dataset of ten scans, i.e., eight tissue-mimicking agar phantoms and two in vivo scans of arteries featuring different motion patterns. We show that *Motion score* had exceptionally high agreement with human annotators in selecting low-motion MS-OPUS frames, the gold standard process, while speeding up the process notably. Moreover, *Motion score* outperformed other methods such as optical flow or cross-correlation by a large margin. Furthermore, we show that frames with low *Motion score* were free of motion-related artefacts and have the optimal spectral quality. Finally, we provide an open-source implementation of *Motion score* as an off-the-shelf tool that can bring an immediate benefit to any clinical or preclinical MS-OPUS analysis pipeline.

**Supplementary Information:**

The online version contains supplementary material available at 10.1038/s41598-025-33753-6.

## Introduction

Technological progress with handheld multispectral optoacoustic-ultrasound (MS-OPUS) imaging has enabled several promising clinical investigations that have imparted label-free visualization of morphological, inflammatory, metabolic and other pathophysiological contrast in breast cancer,^1^ chronic gut inflammation,^2^ thyroid cancer,^3–5^ muscle function,^6^ lymph nodes,^7^ peripheral nerves,^8^ and other conditions. Besides uniquely resolving anatomical or functional features based on optical contrast, optoacoustic (OptA) methods can be seamlessly combined with ultrasound (US) imaging by means of a common ultrasound element array. Collection of tissue optical spectra can be achieved by sequentially recording OptA images at different illumination wavelengths to form multispectral (MS) OptA frames. Each volume element (voxel) in an MS frame has a spectrum representing the combination of the absorption spectra from different light-absorbing molecules present in that voxel, proportional to their concentration. However, motion occurring during the wavelength scan can impact the accuracy of the spectra collected per voxel by mixing contributions collected in a random fashion from adjacent voxels within each spectrum. Such spectral averaging challenges the performance of unmixing algorithms, which seek to identify the concentration of different chromophores in each voxel by identifying their absorption signatures in the spectra collected.

Modern MS-OPUS systems reduce motion artifacts by using fast scanning techniques that acquire tens of wavelengths per second. Nevertheless, due to the high resolution achieved (100–300 micrometers) even small motion occurring on the sub-second time scale can corrupt the data. In pre-clinical multispectral optoacoustic tomography (MSOT) studies, artifacts caused by periodic motion that follows a regular pattern, such as heartbeat and breathing, have been suppressed with the help of frame rejection and motion clustering.^9–11^ However, these methods cannot be translated to clinical handheld scanning, where irregular/non-periodic motion is common. The same applies for methods developed for stationary, bed-based systems with less possibilities for motion than handheld scanners.^12,13^ Faster illumination, i.e., in the kilohertz range,^14^ could shorten the acquisition time and eliminate the influence of motion, however the current best illumination technologies offer wavelength scan rates of up to 100 Hz only, while maintaining a high energy-per-pulse in the range of the tens of milli-joules required in clinical systems.^15,16^

In clinical studies, co-registration of images at different wavelengths onto one reference single-wavelength image has been employed to reduce motion-induced spectral artifacts.^1,17,18^ However, this approach is challenging when aligning images at wavelengths further apart, since the different absorption characteristics of various chromophores may lead to substantial variations of the image appearance at distinct wavelengths. Motion correction based on aligning images according to the skin surface contrast seen on OptA images or US images which are acquired in parallel to OptA images, i.e., co-registered, have also been proposed,^19,20^ but the approaches may not be well suited for aligning pixels that are far from the surface in soft tissues due to plasticity and deformation effects. Equally, speckle tracking in co-registered US images has been applied to correct motion artifacts in coregistered OptA images during acquisition.^21^ However, the speckle tracking can only correct motion artifacts for 50 Hz US frame rates or higher which commercial handheld MS-OPUS systems do not unanimously achieve.^1^ A different approach computes the pairwise cross-correlation between US images corresponding to a single MS frame and uses it to assess the amount of motion in MS-OPUS scans. Then, this latter approach selects frames with the lowest amount of motion as the best candidates for further analysis.^4^ While promising, the accuracy of the method at selecting frames with low motion or with optimal spectral quality has not been validated.

To improve the performance over previous implementations and enable the application in all existing commercial MS-OPUS systems, we introduce *Motion score*, a post-processing algorithm developed to offer automated and accurate quantification of motion in MS-OPUS scans, leading to a reproducible selection of MS frames with the least motion. *Motion score* computes the similarity of the US images acquired over an MS scan and assumes that low similarity implies the presence of motion. *Motion score* is different from previous methodologies in that the similarity between US images is not computed on consecutive frames but on the entire ensemble of US images and therefore avoids bias by offering motion assessment throughout the entire scan. We validate the ability of our algorithm to select optimal frames by comparing the results of the algorithm to results from human annotators, the current standard for motion quantification in MS-OPUS, on a dataset composed of eight scans of an agar tube phantom and two in vivo scans of arteries. We demonstrate that *Motion score* overcomes the limitations of three other selected methods for stationary position detection including the cross-correlation approach previously described,^4^ outperforming them in terms of precision, recall, and mean average precision (mAP). Furthermore, we show on examples of phantom and in vivo scans that the MS frames selected by our algorithm do not suffer from motion-induced artifacts and thus yield the optimal spectral quality required for precise clinical analysis. We discuss how *Motion score* can be readily applied to any existing MS-OPUS systems, since it does not require external tracking or hardware modifications. Finally, we make a ready-to-use, open-source implementation of *Motion score* publicly available together with this paper at www.github.com/jankukacka/optimal_frames.

## Methods

### Multispectral optoacoustic-ultrasound (MS-OPUS) tomography

**Fig. 1:**
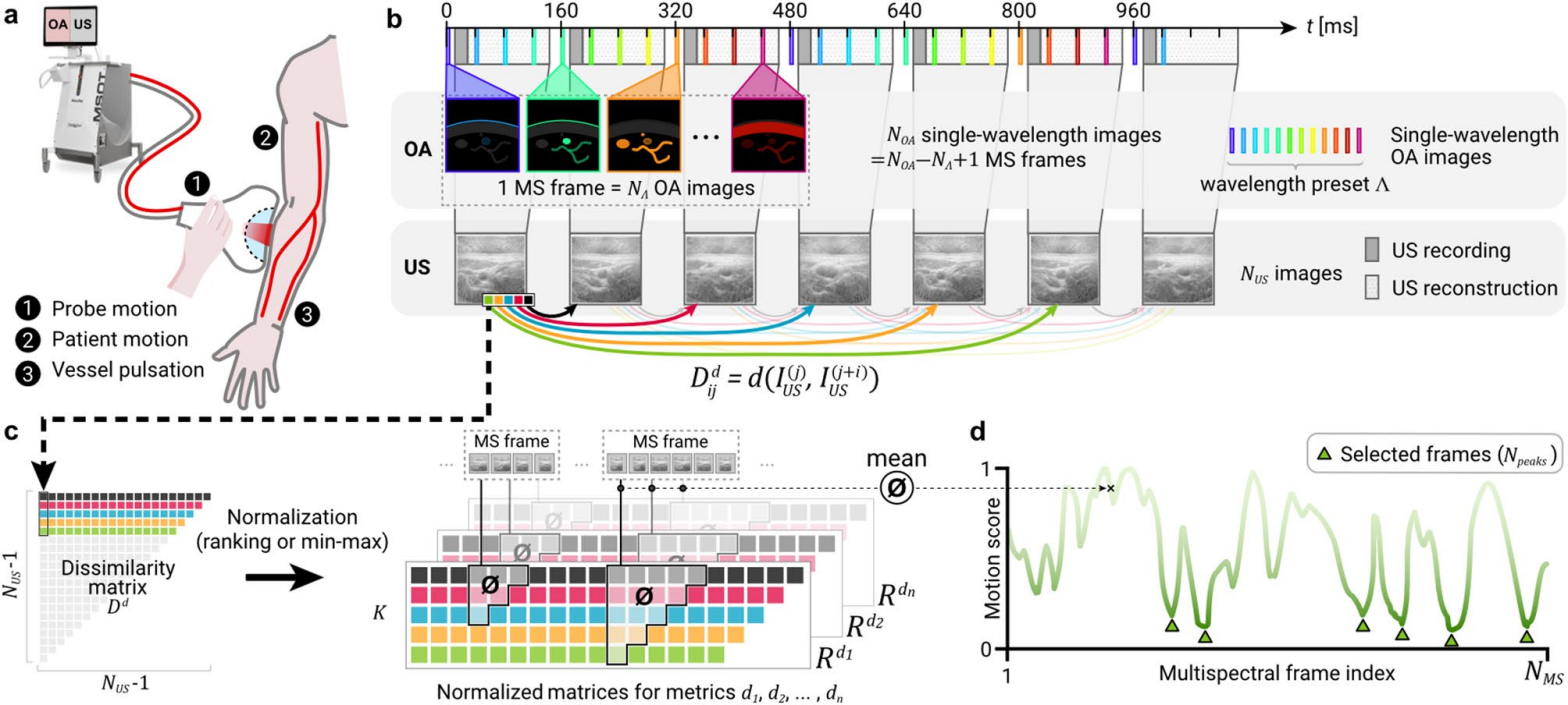
**Multispectral optoacoustic-ultrasound (MS-OPUS) tomography imaging and** ***Motion score*** **computation. (a)** Schematic of the handheld MS-OPUS operation using a *MSOT Acuity Echo*^®^ prototype *(iThera Medical GmbH*,* Munich Germany)* with sources of motion highlighted. **(b)** MS-OPUS scanning scheme. Optoacoustic (OA) and ultrasound (US) images are acquired at different frequencies in an interleaved fashion. Several US images are acquired during recording of a single multispectral (MS) frame. **(c)** Dissimilarities between US images and their subsequent neighbors form the matrix $\:{D}^{d}$. Values of $\:{D}^{d}$ are row-wise ranked to obtain a rank-normalized matrix $\:{R}^{d}$. Multiple dissimilarity measures $\:{d}_{1},\:\dots\:,\:{d}_{n}$ can be combined since the ranking normalization equalizes their scales. The *Motion score* of an MS frame is then computed as the mean of all dissimilarities between its corresponding US images (i.e., the US images that were acquired while recording the MS frame). The number of corresponding US images may vary between MS frames. **(d)** A peak-finding algorithm is applied on the *Motion score* vector of the whole MS-OPUS scan (where a lower *Motion score* means less motion) to identify up to $\:{N}_{peaks}$ stationary MS frames.

An MS-OPUS scanner acquires two streams of data in parallel using a single handheld probe (Fig. 1a). An MS-OPUS scan contains a sequence of US images, $\:{I}_{US}^{\left(j\right)}$, $\:j=1,\dots\:,{N}_{US}$, and a sequence of single-wavelength OptA images, $\:{I}_{OA}^{\left(k\right)}$, $\:k=1,\dots\:,{N}_{OA}$. As shown in Fig. 1b, the OptA images are recorded cyclically at wavelengths from a predefined set $\:{\Lambda\:}$, depending on the system preset. One MS frame consists of $\:{N}_{{\Lambda\:}}=\left|{\Lambda\:}\right|$ consecutive single-wavelength images spanning the whole set of acquired wavelengths: $\:{I}_{MS}^{\left(k\right)}=\left\{{I}_{OA}^{\left(k\right)},\:{I}_{OA}^{\left(k+1\right)},\dots\:{I}_{OA}^{\left(k+{N}_{{\Lambda\:}}-1\right)}\right\}$, $\:k=1,\dots\:,{N}_{MS}={N}_{OA}-{N}_{{\Lambda\:}}+1$. OptA and US images are acquired in an interleaved fashion, and each image is assigned a timestamp $\:{t}_{OA}^{\left(k\right)}$, $\:{t}_{US}^{\left(j\right)}$. Thus, we can define a sequence $\:{\varvec{s}}^{\left(k\right)}\:$of indices of US images corresponding to the $\:k$-th MS frame to be the ordered sequence of indices in the set $\:\left\{i\in\:\mathbb{N}|{t}_{OA}^{\left(k\right)}\le\:{t}_{US}^{\left(i\right)}\le\:{t}_{OA}^{\left(k+{N}_{{\Lambda\:}}-1\right)}\right\}$. In the following text, we omit the frame index ^*(k)*^ where it is obvious from the context.

Other algorithms usually consider MS frames to start with the lowest wavelength image (i.e., $\:k\equiv\:1\:\left(\text{m}\text{o}\text{d}\:{N}_{{\Lambda\:}}\right)$) and end with the highest wavelength. Moreover, MS frames are considered consecutive with no overlap, i.e., single-wavelength images only belong to a single MS frame. We want to highlight that contrary to this usual protocol, we do not require the beginning of an MS frame to be aligned to the lowest wavelength image, while we still sort the images within the MS frame by ascending wavelength before further processing. This has two implications: an MS frame can start at any single-wavelength image in the sequence, and two consecutive MS frames have an overlap of $\:{N}_{{\Lambda\:}}-1$ single-wavelength frames.

### Motion quantification

Let $\:d$ be a dissimilarity function on the space of images (e.g., L_2_-norm). Assuming that two images will be similar in terms of $\:d$ if there is no motion occurring between their acquisitions, we can utilize $\:d$ for motion quantification. However, this assumption does not necessarily hold true for OptA images acquired at different wavelengths, which may display different absorbers, giving them a distinct appearance even in the absence of motion. The US images, on the other hand, have a more consistent appearance than OptA images acquired at different wavelengths. Thus, US images are better suited than OptA images to estimate the motion occurring during an MS-OPUS scan. A naïve approach to compute motion $\:{m}_{0}$ in an MS frame would be to take the mean of dissimilarities between consecutive US frames in its corresponding sequence $\:\varvec{s}$:

1 $$\:\begin{array}{c}{m}_{0}=\frac{1}{\left|\varvec{s}\right|}\sum\:_{j={\varvec{s}}_{1}}^{{\varvec{s}}_{\left|\varvec{s}\right|-1}}d\left({I}_{US}^{\left(j\right)},{I}_{US}^{\left(j+1\right)}\right).\end{array}$$

This approach has two limitations. First, it cannot differentiate between (acceptable) jitter and (much more problematic) steady drift. Second, it suffers from biases of the chosen dissimilarity function $\:d$ towards penalizing certain types of motion more than others. To overcome these limitations, we propose *Motion score*, a motion quantification framework for MS-OPUS.

### Motion score

Figure 1c outlines the *Motion score* computation. Let $\:{D}^{d}\in\:{\mathbb{R}}^{{\left({N}_{US}-1\right)}^{2}}$ be a matrix of dissimilarities between US images within one MS-OPUS scan measured by $\:d$:

2 $$\:\begin{array}{c}{D}_{ij}^{d}=\left\{\begin{array}{cc}d\left({I}_{US}^{\left(j\right)},{I}_{US}^{\left(j+i\right)}\right)&\:\text{i}\text{f}\:i+j\le\:{N}_{US}\\\:\text{u}\text{n}\text{d}\text{e}\text{f}\text{i}\text{n}\text{e}\text{d}&\:\text{e}\text{l}\text{s}\text{e}\end{array}\right..\end{array}$$

Element $\:j$ in row $\:i$ corresponds to the dissimilarity between the $\:j$-th image and its $\:i$-th subsequent image in the US image sequence. Elements under the anti-diagonal are undefined since they correspond to subsequent images, which would be beyond the end of the sequence. In practice, only the first $\:K=\underset{k}{\text{max}}\left|{\varvec{s}}^{\left(k\right)}\right|$ rows of the matrix $\:{D}^{d}$ must be computed, because a MS frame comprises at most $\:K$ US images which need to be compared to estimate motion per frame. Furthermore, let $\:{R}^{d}$ and $\:{N}^{d}$ be matrices with the same shape as $\:{D}^{d}$ and elements in the range [0,1], i.e., are normalized, allowing the comparison of different dissimilarity measures. We define $\:{R}^{d}$ as a matrix of normalized row-wise ranks of the elements of $\:{D}^{d}$, $\:{R}_{ij}^{d}=\left(\text{r}\text{a}\text{n}\text{k}\:\text{o}\text{f}\:{D}_{ij}^{d}\:\text{i}\text{n}\:{D}_{i}^{d}\right)/\left({N}_{US}-i-1\right)$, where the rank is defined as the position in the sorted row and not in the mathematical sense. $\:{N}^{d}$ is a matrix of row-wise min-max normalized elements of $\:{D}^{d}$, $\:{N}_{ij}^{d}=\left({D}_{ij}^{d}-\underset{k}{\text{min}}{D}_{ik}^{d}\right)/\left(\underset{k}{\text{max}}{D}_{ik}^{d}-\underset{k}{\text{min}}{D}_{ik}^{d}\right)$. Finally, let $\:{\Delta\:}=\left\{{d}_{1},\dots\:,{d}_{n}\right\}$ be a set of dissimilarity measures. Then we define the ranked *Motion score*, $\:{m}_{R}^{{\Delta\:}}$, and the normalized *Motion score*, $\:{m}_{N}^{{\Delta\:}}$, for an MS frame with a corresponding sequence of US image indices $\:\varvec{s}$ as:

3 $$\:\begin{array}{c}{m}_{R}^{{\Delta\:}}=\frac{2}{\left|{\Delta\:}\right|{\left|\varvec{s}\right|}^{2}}\sum\:_{d\in\:{\Delta\:}}\sum\:_{i=1}^{\left|\varvec{s}\right|}\sum\:_{j={\varvec{s}}_{1}}^{{\varvec{s}}_{\left|\varvec{s}\right|-i}}{R}_{ij}^{d}\:,\end{array}$$

4 $$\:\begin{array}{c}\:\:{m}_{N}^{{\Delta\:}}=\frac{2}{\left|{\Delta\:}\right|{\left|\varvec{s}\right|}^{2}}\sum\:_{d\in\:{\Delta\:}}\sum\:_{i=1}^{\left|\varvec{s}\right|}\sum\:_{j={\varvec{s}}_{1}}^{{\varvec{s}}_{\left|\varvec{s}\right|-i}}{N}_{ij}^{d}\:.\end{array}$$

An example for the *Motion score* computation is provided in the Supplementary (see Supplementary Information – *Motion score* – computation example).

### Frame selection

Figure 1d shows a motion vector $\:\varvec{m}$ of length $\:{N}_{MS}$ obtained by computing the motion of all MS frames in a scan. To automatically select the best stationary frames, a peak-finding algorithm “find_peaks” from the SciPy library (v1.8.0) ^22^ was used to identify local minima of the vector $\:\varvec{m}$. The detected peaks with a minimal distance $\:{d}_{min}\:$were sorted by ascending values and the first $\:{N}_{peaks}$ were taken. The minimal peak distance $\:{d}_{min}$ and the number of detected peaks $\:{N}_{peaks}$ are both hyperparameters. We observed empirically that $\:{d}_{min}=\lfloor{N}_{MS}/20\rfloor$, i.e., equal to 1/20 of the scan length $\:{N}_{MS}$, and $\:{N}_{peaks}=5$ are reliable values for the MS-OPUS scans used in the development of the algorithm. However, both hyperparameter values depend on the number of US images acquired per frame $\:K$, the average scan length and the number of expected stationary positions in a scan, i.e. the scanning protocol. For this reason, the parameters should be adapted accordingly. Increasing the number of detected peaks $\:{N}_{peaks}$ decreases the risk of missing a desired location but increases the chance of false positives. Equally, a minimal length distance between peaks smaller than the number of US images per frame might result in the detection of numerous stationary frames with overlapping single-wavelength OptA images but large values might cause the risk of missing desired locations. Any other parameters of algorithms employed were left at their default settings.

### Validation

Our first goal was to validate the ability of *Motion score* to automatically select stationary frames in MS-OPUS scans and to compare its performance to other metrics.

### MS-OPUS setup

We used two datasets acquired with *MSOT Acuity Echo*^®^ scanners (iThera Medical GmbH, Munich, Germany) with a wavelength preset $\:{\Lambda\:}=\left\{700\:\text{n}\text{m},\:710\:\text{n}\text{m},\dots\:,970\:\text{n}\text{m}\right\},\:{N}_{{\Lambda\:}}=28.$ The acquisition frequency was 25 Hz and 6.25 Hz for the OptA single-wavelength images and the US images, respectively. The datasets include scans of both a tissue-mimicking phantom and human subjects. The authors confirm that all human subject research procedures and protocols are exempt from review board approval.

**Dataset 1** (10 scans) was obtained using a probe with 256 transducers (5.2 MHz central frequency, 1.3 cm chord length) arranged in a 4 cm wide 125° arc filled with a gel pad for acoustic coupling. Various types of probe motion (linear, rotational, slow and fast, steady and jerking, etc.) are captured in this dataset. The first eight scans were conducted on a tissue-mimicking agar phantom with various optical absorbers (see Supplementary Information – Phantom manufacturing). Another two scans targeted the carotid artery of a healthy volunteer. The duration of the scans was between 35 s and 120 s. The acquisition times per scan exceed common clinical scanning durations for a single location. Instead, motion affected, and stationary frames were deliberately acquired together in a single scan. In total, the dataset contains 11.6 min of recorded data.

**Dataset 2** (1 scan) was obtained with an older prototype using a probe with 256 transducers (4 MHz central frequency, 2.6 cm chord length) arranged in a 6 cm wide 145° arc filled with heavy water. The scan, showing the radial artery from a healthy volunteer, resembles a typical MS-OPUS clinical acquisition. We selected this dataset to demonstrate the efficiency of our method on a generic acquisition and to showcase how motion can distort spectra of in vivo MSOT images.

### Stable position annotation

To derive the ground truth annotations, three separate human experts selected all stationary frame ranges in the US videos in Dataset 1. Their annotations were summed to assign a score 0–3 to each US image; MS frames were assigned the mean score of all their corresponding US images, and scores ≥ 1.5 were considered stationary. For quantitative evaluation, the task of stationary position selection was framed as an event detection problem. Any uninterrupted sequence of stationary MS frames was considered to be a target event and selecting any MS frame within such a sequence was considered a true positive prediction (TP). On the other hand, selecting a frame outside a target event (stationary sequence) was false positive prediction (FP) and any event not covered by any prediction was counted as a false negative (FN).

## Motion quantification

To compare *Motion score*’s motion quantification performance, motion in all scans of Dataset 1 was quantified using both normalized *Motion score* (Eq. (3)) and ranked *Motion score* (Eq. (4)). Our two methods were compared to three methods computing a simple mean dissimilarity (Eq. (1)) by means of cross correlation, zeroed-normalized cross-correlation (ZNXC), or optical flow (see Supplementary Information – Dissimilarity measures).

### Evaluation metrics

Three parameters, i.e., precision, recall, and mAP, were employed to evaluate the accuracy of the our two and the three reference motion quantification methods. Precision is defined as the ratio of true positives in all predicted positions (TP/TP + FP). Recall is defined as the fraction of target events that were correctly detected, computed as the ratio of unique true positives (TP_1_) in all positives (TP_1_/TP_1_ + FN). TP_1_ is a subset of TP allowing at most one predicted position per target event. For computing precision and recall, $\:{N}_{peaks}$ was set to match the number of stationary positions identified by the human annotators in each scan. Since this number is generally unknown, the area under the precision-recall curve for all values of $\:{N}_{peaks}$ was also computed for each scan (average precision), and the mean over all scans was computed to obtain the mAP.

### Spectral quality evaluation

Our second goal was to demonstrate that the frames selected by the *Motion score* algorithm indeed suffer from fewer motion-related artifacts and thus have better spectral quality. Here, we assume that a measured spectrum in a pixel should be a linear combination of the absorption spectra of the chromophores present in that pixel. This linear combination can be computed by linear spectral unmixing of the form:

5 $$\:\begin{array}{c}C={\underset{C\ge\:0}{\text{argmin}}\parallel{I}_{MS}-CW\parallel}_{F}\end{array}$$

Here, $\:{I}_{MS}\in\:{\mathbb{R}}^{p\times\:\left|{\Lambda\:}\right|}$ is an MS frame with the spectra of its $\:p$ pixels arranged in rows, $\:C\in\:{\mathbb{R}}^{p\times\:k}$ is a matrix of unmixing coefficients, and $\:W\in\:{\mathbb{R}}^{k\times\:\left|{\Lambda\:}\right|}$ is a matrix of $\:k$ spectral components, which are either fixed known spectra of pre-defined absorbers or optimized together with $\:C$ by a data-driven blind unmixing procedure. $\:C\ge\:0$ denotes a non-negativity constraint on the elements of $\:C$.

Processes violating the linear decomposition assumption cause spectral corruption. Aside from spectral coloring resulting from uneven light fluence in the tissue, motion is the main source of spectral corruption. Spectral corruption can be quantified as the magnitude of residuals after linear spectral unmixing, i.e., the amount of signal that cannot be explained by a linear mixing model. The residuals are expected to be low if the measured spectra can be decomposed (unmixed) into a linear combination of several spectral components corresponding to the absorbers in the image. However, if the spectrum is corrupted by motion, it will contain irregularities that cannot be unmixed into the expected components and the unmixing residuals will be high. The unmixing residual (error) is computed as the L_2,1_-norm (sum of L_2_-norms over the matrix columns) of the residuals relative to the norm of the image:

6 $$\:\begin{array}{c}E\left({I}_{MS},C,W\right)=\frac{{\parallel{\left({I}_{MS}-CW\right)}^{T}\parallel}_{\text{2,1}}}{{\parallel{I}_{MS}^{T}\parallel}_{\text{2,1}}}\:.\:\end{array}$$

In our experiments, two types of spectral unmixing were used. For phantom scans from Dataset 1, blind unmixing by non-negative matrix factorization (NMF) was used with $\:k=4$ to match the number of expected absorbers in the images (contrast agent, agar, psyllium husks, and probe membrane). For the clinical scan from Dataset 2, standard linear unmixing according to Eq. (5) was used, with the spectra of oxy- and deoxyhemoglobin, lipids, and water.

## Results

### *Motion score* accurately identifies stationary frames in MS-OPUS scans

**Fig. 2:**
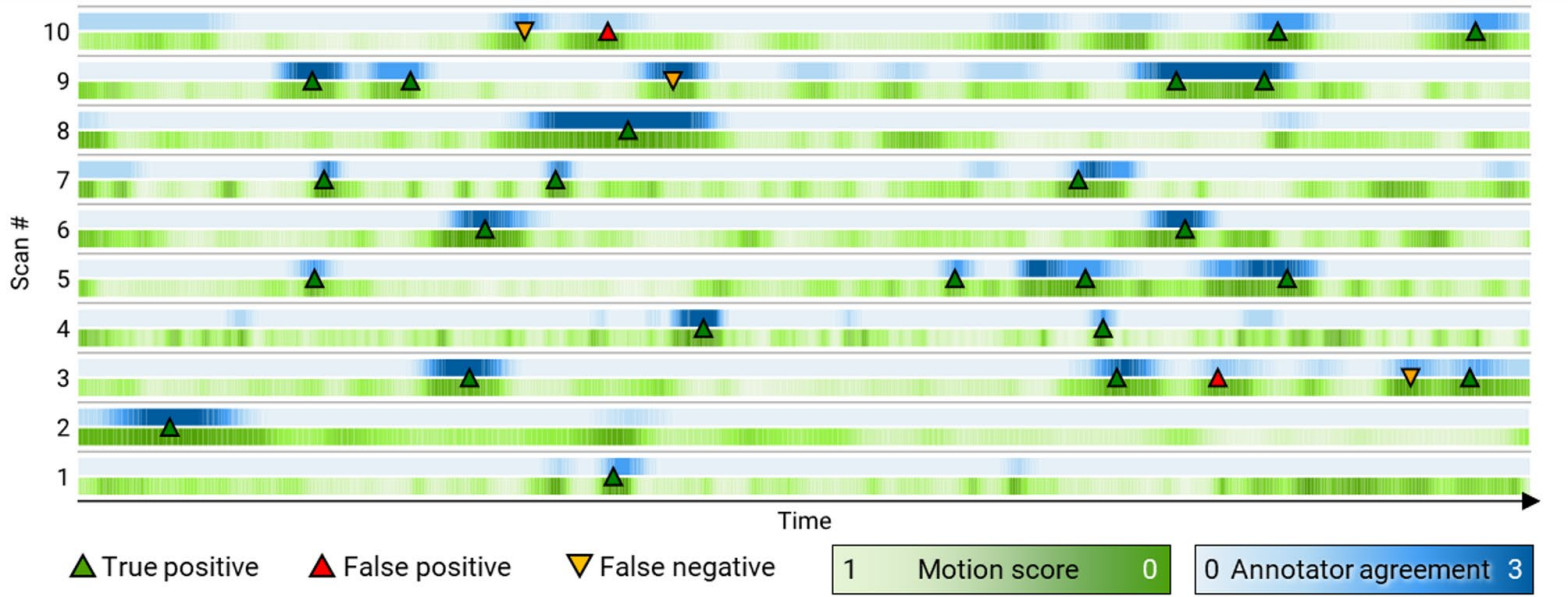
Stationary position detection evaluation on multispectral optoacoustic-ultrasound (MS-OPUS) scans. Three human annotators reviewed US videos of ten MS-OPUS scans (Dataset 1) and identified 25 stationary positions (blue). *Motion scores* of those scans were also computed (green) and the most optimal frames were selected (n=$\:{N}_{peaks})$, such that $\:{N}_{peaks}$ matches the number of true stationary positions identified by the human annotators. The selected frames are marked by green (true positive) and red triangles (false positive). Stationary positions missed by our algorithm (false negatives) are marked by yellow upside-down triangles.

*Motion score* has shown nearly perfect agreement with human annotators when finding stationary positions in MS-OPUS scans. Three annotators identified in total of *n* = 25 stationary positions in the 10 scans of Dataset 1 (1–4 positions per scan). On average, the annotators needed 8.5 min to annotate 1 min of US video. *Motion score* (ranked; $\:{\Delta\:}=\{ZNXC,\:SSIM\}$) was applied to select a number of frames in each scan ($\:{N}_{peaks})$ to match the ground truth number identified by the three annotators. Figure 2 shows that of these 25 selected frames, 23 were located within 22 of the true stationary positions (92% precision, 88% recall). Furthermore, the two false positive frames were in positions that were labeled stationary by at least one annotator (red triangles). Similarly, the three false negative locations (yellow triangles) were also aligned with *Motion score* local minima, but their *Motion score* values were higher than in the 25 selected frames. Figure 2 also shows qualitatively that Motion score identifies local low-motion regions in the scans, which were, however, not detected as stationary positions, because the specified number of stable positions in each scan was already detected. Since the number of stable positions per scan is generally unknown, we also evaluated the mAP of stationary position detection, which measures the precision-recall over all values of $\:{N}_{peaks}$, and obtained a high mAP of 91.67%. Table 1 shows a performance comparison of *Motion score* to other metrics. *Motion score* outperformed naïve approaches (rows 1–3), where only directly consecutive US images were compared, by a wide margin. Unnormalized cross-correlation and optical flow performed particularly poorly. Finally, the ranked *Motion score* achieved a better mAP than the normalized version (+ 3.3% points).

**Table 1:** Stationary position detection performance. Performance of the five methods for identifying stationary positions evaluated on dataset 1. Abbreviations: ZNXC—zeroed normalized cross-correlation; SSIM—structural similarity; mAP—mean average precision.

Method	Precision	Recall	mAP
Cross-correlation	4/25	5/25	22.50%
Zeroed normalized cross-correlation	20/25	19/25	74.17%
Optical flow (FlowNet2)	17/25	15/26	58.33%
*Motion score* normalized (ZNXC + SSIM)	22/25	21/25	88.33%
*Motion score* ranked (ZNXC + SSIM)	**23/25**	**22/25**	**91.67%**

Low *Motion score* correlates with optimal spectral quality.

**Fig. 3:**
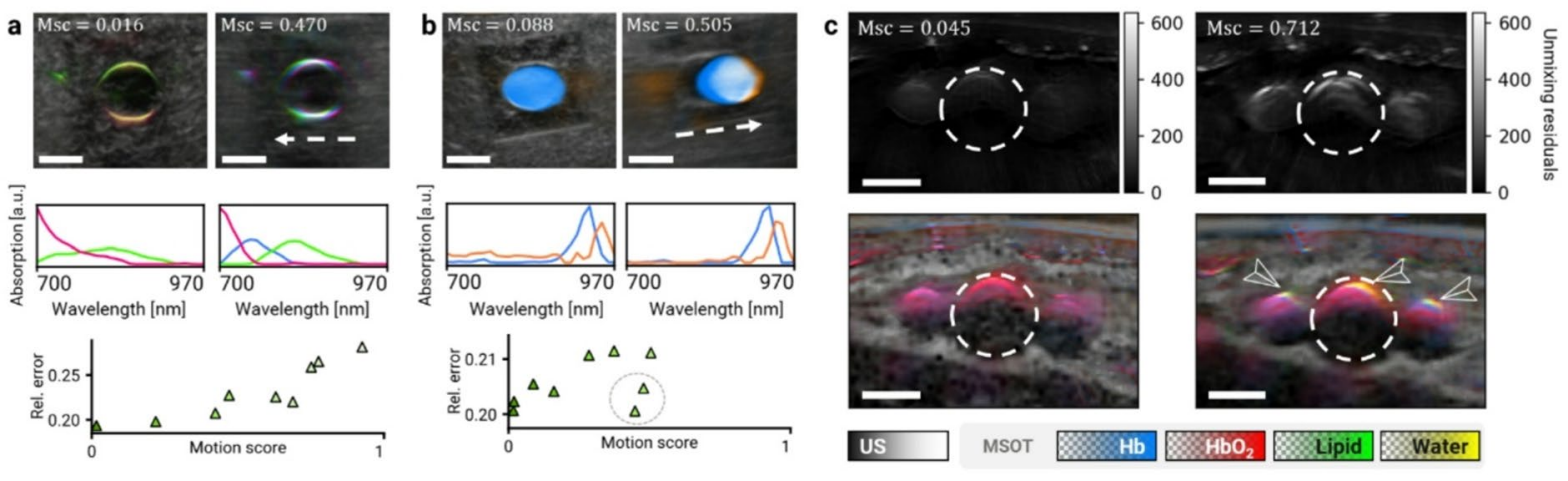
**Lower** ***Motion score*** **leads to fewer motion artifacts and better spectral quality. (a)** Scans of a cavity filled with oil red O organic dye embedded in an agar phantom without motion (left) and with motion along the dashed arrow (right). Grayscale ultrasound (US) images are overlaid with color-coded concentrations of components identified via non-negative matrix factorization (NMF). Below, their spectra are shown in matching colors. Bottom: relationship between *Motion score* and relative NMF unmixing residuals (4 components). **(b)** The same analysis as in (a) for a scan of a cavity filled with olive oil. The gray circle in the bottom scatterplot denotes frames in which motion was parallel to the tubular cavity and did not cause considerable spectral corruption. **(c)** Scans of a radial artery (dashed circles) and concomitant veins without motion (left) and with motion (right). Top row: Residuals after linear unmixing (both using the same color scale) show that motion causes spectral artifacts around the blood vessel edges that cannot be properly unmixed. Bottom row: visualization of unmixing coefficients of Hb (blue), HbO_2_ (red), water (yellow), and lipid (green). Motion-related spectral artifacts appear as additional stripes of water and fat signal around the artery and veins (arrows). Scale bars: 5 mm. Abbreviations: Msc—Motion score; Hb—deoxyhemoglobin; HbO_2_—oxyhemoglobin.

We observed that stationary MS frames, i.e., frames with low *Motion score*, did not suffer from motion-related artifacts. Figure 3 shows examples of motion-related artifacts in two representative phantom scans with good OptA contrast from Dataset 1 (see Supplementary Information – Phantom manufacturing) and the in vivo scan from Dataset 2. The phantom scans depict agar cavities filled with absorbers having distinct spectral appearances: oil red O organic dye (Fig. 3a) with a spectrum whose intensity gradually decreases with increasing wavelength, and olive oil (Fig. 3b) with a narrow absorption peak at 930 nm. Motion corrupts the spectra in both cases, regardless of the absorber. The images on the left, having low *Motion scores*, represent stationary frames, whereas the images on the right show frames corrupted by motion along the dashed arrows. In scans of both phantoms, blind unmixing detected either spectral components with a different shape or different spatial distribution in the image (middle row) in the frames with high *Motion scores* compared to stationary frames. In Fig. 3a, the absorption spectrum in the stationary frame was decomposed into two components representing variations in light fluence, colocalized around the cavity edges. In the moving frame, three components were identified with disjoint absorption peaks at 700 nm, 760 nm, and 820 nm, appearing in the image as three crescents horizontally shifted along the motion direction. In Fig. 3b, two similar spectral components were identified in both images showing contrast throughout the entire cavity—component 1 (blue) fitting the true absorption peak of olive oil at 930 nm, and component 2 (orange) with a peak shifted towards 950 nm. In the stationary frame, only component 1 is present in the cavity, whereas the moving frame contains two, partly disjointed discs of the two components. In both cases, unmixing results from images affected by motion defy the true phantom composition, demonstrating the unsuitability of such frames for spectral analysis.

Furthermore, we observed that low *Motion score* indicates good spectral quality. To measure the spectral quality, we evaluated the unmixing residuals. The scatter plots in the bottom rows of Figs. 3a, b show the relationship between *Motion score* and the unmixing residuals. Although there were frames with low unmixing residuals despite high *Motion score* (Fig. 3b, gray circle—these frames contain motion along the tubular cavity and hence the position of the absorber cross-section remains stable), there were no frames with low *Motion score* and high unmixing residual, demonstrating that a low *Motion score* is a good indicator of optimal spectral quality.

Finally, we verified that *Motion score* is effective at selecting stationary frames with good spectral quality in a generic clinical MS-OPUS acquisition. Figure 3c demonstrates motion-induced spectral artifacts in a clinical scan of a radial artery (dashed circle) from a healthy volunteer (Dataset 2). The upper row shows unmixing residuals (same color scale) for a stationary (left) and moving (right) frame. The bottom row shows visualization of the unmixed chromophores (oxy- and deoxyhemoglobin, lipid, and water) over a grayscale US image. Whereas only hemoglobin is detected inside the blood vessels in the stationary frame (as expected), unmixing of the motion-corrupted frame contains erroneous signals of lipids and water around the blood vessel edges (arrows).

## Discussion

In this work, we introduced *Motion scor*e as a post-processing algorithm for motion quantification robust against different kinds of motion and automated stationary frame selection in MS-OPUS scans. We have validated the accuracy of our method and demonstrated its superiority to other motion quantification approaches previously introduced. Furthermore, we have shown that frames selected by *Motion score* have fewer motion-related artifacts, yielding optimal spectral quality.

The selection of stationary frames, i.e., frames least affected by motion, is critical in order to fundamentally improve spectral performance, since even after correction, frames with high motion effects can suffer from artifacts and errors that cannot be successfully restored. In the phantom scans, additional, false absorbers with shifted absorption peaks were spectrally unmixed in motion corrupted MS-OPUS frames. The different appearance of the phantom insertions in Fig. 3a, b are independent of motion as they appear both in motion affected and stationary frames. We assume the different appearance is caused by the organic dye depositing on the insertion walls. We could not verify this hypothesis as the phantom had to be disposed of before the analysis could be performed. In the scans of the radial artery, significant spectral unmixing errors in terms of unrealistic increased water and lipid signals around the edges of the blood vessel were observed in frames with high *Motion score*. Such unmixing errors negatively impact the accuracy of clinical MS-OPUS applications and may hurt the reliability of evaluations of post-prandial lipemia^23^ or carotid plaques.^24^ On the other hand, frames selected using *Motion score* showed consistently good spectral quality at a level required for precise molecular analysis.

Our motion quantification benchmark showed that *Motion score* outperformed all other methods we evaluated. We hypothesize that the poor performance of the naïve approach in which only the mean of a single dissimilarity metric for neighboring US images in a single MS frame is computed (see Eq. (1)) is caused by two issues which are tackled by the algorithm. First, *Motion score* considers the distances between all pairs of US images within one MS frame, enabling the penalization of undesirable continuous drifting motion. Second, *Motion score* alleviates the bias resulting from the selection of only one metric by combining any number of dissimilarity measures using the rank-score or min-max normalization. Normalization equalizes different value scales and makes the measures comparable. The rank-score normalization is less sensitive to outliers than min-max normalization, as an image pair with exceptionally high dissimilarity cannot outweigh the relative differences between other pairs since only the order of the values is considered. Ranking in *Motion score* has an additional advantage when comparing MS frames with different numbers of corresponding US frames (which happens unless $\:\exists\:k\in\:\mathbb{N}:k/{f}_{US}={N}_{{\Lambda\:}}/{f}_{OA}$), because US frames with different number of images would be compared. Since the US images acquired further apart tend to have a higher dissimilarity, their perceived motion would also tend to be higher. Using any of both normalization techniques presented eliminates this bias. These advantages, together with better performance in our experiment, make the ranked *Motion score*, i.e., *Motion score* with rank-score normalization, the preferable variant.

In this study, we employed human assessment as our reference standard to quantify motion in MS frames. Obvious drawbacks of this approach are the lack of reproducibility and qualitative nature. A quantitative measure of motion could be obtained using skin tattoos^25^ or optical tracking,^26^ however, these methods only track the movement of the probe but not the patient or the internal tissue. Human annotation, on the other hand, considers every source of motion that affects image appearance, which is desired in our scenario. Furthermore, to reduce interobserver variance, our experiment aggregated evaluation from three independent annotators. As such, human assessment was indeed a reasonable choice of a reference standard. Moreover, the US frame rate of the commercial MS-OPUS imaging device employed was only 6.25 Hz limiting the ability to track motion in consecutive US images accurately. Nevertheless, our experiments showed that Motion score succeeds in distinguishing motion affected and stationary frames and consequently leading to supreme spectral data quality.

*Motion score* is a relatively simple algorithm, yet robust against different kinds of motion possible and observed in clinical MS-OPUS imaging. In this work, we validated *Motion score* using scans acquired with a 28-wavelength preset, where most MS frames had 9 or 10 corresponding US images. Possible performance deterioration could occur in scans with fewer wavelengths (and fewer US images per MS frame). This could be remedied by simply combining several MS frames together for the purpose of motion quantification. On the other hand, for scans with more than 28 wavelengths acquired per MS frame, the frame acquisition time increases, and it becomes less likely that tissue or operator motion can be avoided. Hence, the number of wavelengths per MS frame should be limited to the number of wavelengths necessary for multispectral analysis. However, possible performance decrease compared to our validation caused by *Motion score* should be considered for less than 28 wavelengths. Due to its generic nature, *Motion score* can be extended by including additional similarity measures aside from the ZNXC and SSIM measures shown herein. On the other hand, adding poorly performing metrics, such as unnormalized cross-correlation or optical flow computed by a general purpose pre-trained FlowNet2 (see Table 1), would reduce the algorithm’s performance and should be avoided.

Despite its motion quantification capabilities, *Motion score* cannot be used as a universal quality measure in MS-OPUS scans, since the metric produced only relates to the motion in a particular scan, as it is affected by tissue and operator motion. Furthermore, *Motion score* cannot be used for live feedback to the MS-OPUS operator since it is necessary to first acquire entire wavelength scans before *Motion score* can be computed. It should be investigated in the future to enable real-time motion quantification or correction during scanning. Nevertheless, the automatic selection of the stationary frames eliminates the requirement for manual selection, which is non-reproducible and time consuming. In our validation, a human annotator needed eight times longer than the length of the scan itself to select good frames. Therefore, *Motion score* provides a clear and quantitative justification for its use in standardized MS-OPUS analysis.

Moreover, *Motion score* could even lower operator burden, making MS-OPUS ultimately more clinically practical. Standard procedure requires operators to examine patients, keeping the probe stationery at positions of interest, and initiating data acquisition manually, adding an additional risk of operator motion. Instead, the operator could acquire the whole scan of patients and only keep the probe stationery at positions of interest. Using *Motion score*, the positions of interest will be automatically identified in post-processing allowing more continuous scanning by the operator with less sources for motion.

Overall, *Motion score* improves spectral quality in MS-OPUS and can therefore play a role in the standardization of MS-OPUS datasets and quality control operations, quantifying inter- and intra-operator variability. Our open-source implementation can immediately be used off-the-shelf in any MS-OPUS analysis pipeline. Altogether, this work paves the way for better utilization of MS-OPUS for clinical applications, improving the accuracy and reliability of the technique.

## Supplementary Information

Below is the link to the electronic supplementary material.

Supplementary Material 1

## Data Availability

The source code of Motion score is available online under a MIT license: www.github.com/jankukacka/optimal_frames. Validation datasets 1 (phantom) and 2 (healthy volunteer) including the US video of dataset 1 are not publicly available but may be obtained from the corresponding author upon reasonable request.

## Abbreviations

FN: False negative
FP: False positive
mAP: Mean average precision
MS: Multispectral
MS-OPUS: Multispectral optoacoustic-ultrasound tomography
NMF: Non-negative matrix factorization
OptA: Optoacoustic
SSIM: Structural similarity
TP: True positive
TP_1_: Unique true positive
US: Ultrasound
ZNCX: Zeroed-normalized cross-correlation
