# Supplementary material for: Motion score for spectral quality control of optoacoustic-ultrasound data
Source: Sci Rep. 2026 Jan 10;16:1325. doi: 10.1038/s41598-025-33753-6 (PMC12796235; doi:10.1038/s41598-025-33753-6)
Supplement: Supplementary file 1 — Supplementary Material 1 [file 41598_2025_33753_MOESM1_ESM.pdf]

Motion score for spectral quality control of optoacoustic-ultrasound data

Jan Kukačka[1,2], Maximilian Bader[1,2], Dominik Jüstel[1,2,3], Vasilis Ntziachristos[1,2,4,5,6,7,✉]

[1] Chair of Biological Imaging, Central Institute for Translational Cancer Research (TranslaTUM), School of Medicine and Health & School of Computation, Information and Technology, Technical University of Munich, Munich, Germany
[2] Institute of Biological and Medical Imaging, Bioengineering Center, Helmholtz Zentrum München, Neuherberg, Germany
[3] Institute of Computational Biology, Computational Health Center, Helmholtz Zentrum München, Neuherberg, Germany
[4] Foundation for Research and Technology Hellas (FORTH), Heraklion, Greece; Institute of Electronic Structure and Laser (IESL)
[5] Munich Institute of Biomedical Engineering (MIBE), Technical University of Munich, Garching b. München, Germany
[6] Munich Institute of Robotics and Machine Intelligence (MIRMI), Technical University of Munich, Munich, Germany
[7] DZHK (German Centre for Cardiovascular Research), partner site Munich Heart Alliance, Munich, Germany.
✉ Corresponding author. Email: bioimaging.translatum@tum.de,
Address: Chair of Biological Imaging, Technical University of Munich, Ismaninger Straße 22, D-81675 Munich, Germany

Supplementary Information

Phantom manufacturing

A tissue-mimicking agar phantom (Figure S1) containing various acoustic and optical imaging targets was manufactured using the following procedure. Five batches of phantom material were mixed according to the quantities listed in Table S1 and warmed up to 90°C while continuously stirring to avoid the creation of air bubbles. The heated mixture was poured into a box of size 20x30x20 cm and allowed to cool down and solidify for 30 min before the next layers were added. Materials of varying density were used for different layers to mimic acoustic interfaces inside the tissue. Psyllium husks were added to create acoustic inhomogeneities providing texture on US images. No other optical scatterers or absorbers were admixed to the phantom material, resulting in a semi-opaque appearance. Three types of objects made of denser agar (same as layer 1, see Table S1) were embedded into the phantom body: (1) agar blocks of various shape and size, (2) cylinders of denser agar containing indocyanine green (15 µg/ml), (3) blocks of denser agar with cylindrical cavities filled with optical absorbers (olive oil, oil red O organic dye). Whereas all three types were visible on the ultrasound, only targets (2) and (3) were visible in the OptA images. The locations of all inserted materials were noted during phantom preparation.

Table S1: Composition of the layers of the tissue-mimicking agar phantom.

Layer	Deionized water (l)	Agar (g)	Glycerol (ml)	Psyllium (g)
1	1.0	36.0	50	5.8
2	0.5	7.5	25	5.0
3	0.5	10.0	25	7.0
4	0.5	12.0	25	5.0
5	0.5	10.0	25	5.0

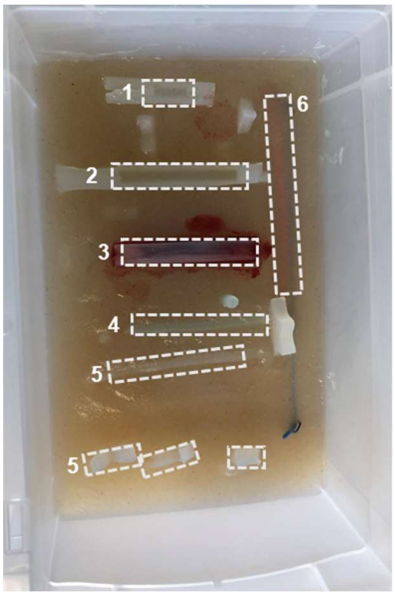

Figure S1: Tissue-mimicking phantom used to collect scans 1–8 from Dataset 1. Dashed lines mark following insertions: (1) cavity with sunflower oil, (2) cavity with olive oil, (3) cavity with oil red O organic dye, (4) cylinder of agar with indocyanine green, (5) stiff agar, (6) rubber tubes filled with water. The photo captures the phantom before the addition of the last agar layer.

Dissimilarity measures

The following dissimilarity measures were used in this work: cross-correlation (XC), zeroed-normalized cross-correlation (ZNXC), structural similarity (SSIM) [1], and optical flow. XC and ZNXC were computed as

$$XC\left(I_{US}^{(i)}, I_{US}^{(j)}\right) = -I_{US}^{(i)} \cdot I_{US}^{(j)}, \tag{S1}$$

$$ZNXC\left(I_{US}^{(i)}, I_{US}^{(j)}\right) = -\left(\frac{I_{US}^{(i)} - \mu^{(i)}}{\sigma^{(i)}}\right) \cdot \left(\frac{I_{US}^{(j)} - \mu^{(j)}}{\sigma^{(j)}}\right), \tag{S2}$$

where I_{US} is a vector of pixels contained in an ultrasound image and $\mu^{(i)}$, $\sigma^{(i)}$ are the mean and the sample standard deviation of the image pixel intensities, respectively.

Structural similarity: Implementation of scikit-image (v0.19.2) [2] was used with default parameters. Since *Motion score* requires distance instead of similarity metrics, the negative value of the SSIM was used.

Optical flow: Pre-trained FlowNet2 [3] (weights obtained from www.github.com/NVIDIA/flownet2-pytorch) was applied on pairs of ultrasound images resized to 192x192 pixels to compute the optical flow between them. The resulting vector field was converted to a scalar by taking the mean of L_2 norms of the displacement vectors at every pixel.

Motion score – computation example

Let the total number of US images in a scan be $N_{US} = 5$ and sequences of US image indices corresponding to MS frames $k \in \{1,2,3\}$ be

$$\boldsymbol{s}^{(1)} = \{1,2\}, \qquad \boldsymbol{s}^{(2)} = \{2,3,4\}, \qquad \boldsymbol{s}^{(3)} = \{4,5\}. \tag{S3}$$

Then, the maximum number of US images corresponding to a MS frame is $K = 3$. The corresponding dissimilarity matrix $D^1 \in \mathbb{R}^{4^2}$ for an arbitrary dissimilarity measure d_1 may then be

$$D^1 = \begin{pmatrix} 1 & 5 & 3 & 4 \\ 2 & 10 & 7 & \\ 50 & 1 & & \\ & & & \end{pmatrix}. \tag{S4}$$

Then, matrices R^1 and N^1 are

$$R^1 = \begin{pmatrix} 0 & \frac{3}{3} & \frac{1}{3} & \frac{2}{3} \\ 0 & \frac{2}{2} & \frac{1}{2} & \\ 1 & 0 & & \end{pmatrix}, \tag{S5}$$

$$N^1 = \begin{pmatrix} 0 & 1 & \frac{1}{2} & \frac{3}{4} \\ 0 & 1 & \frac{5}{8} & \\ 1 & 0 & & \end{pmatrix}. \tag{S6}$$

Given we only compute *Motion score* for one metric $\Delta = \{d_1\}$, the ranked *Motion score*, m_R^Δ, and the normalized *Motion score*, m_N^Δ, for the three MS frames are

$$m_R^{\Delta^{(1)}} = \frac{2}{2^2}(R_{11}^1) = 0, \qquad m_R^{\Delta^{(2)}} = \frac{2}{3^2}(R_{12}^1 + R_{22}^1 + R_{13}^1) = 0.52, \qquad m_R^{\Delta^{(3)}} = \frac{2}{2^2}(R_{14}^1) = 0.34. \tag{S7}$$

$$m_N^{\Delta^{(1)}} = \frac{2}{2^2}(N_{11}^1) = 0, \qquad m_N^{\Delta^{(2)}} = \frac{2}{3^2}(N_{12}^1 + N_{22}^1 + N_{13}^1) = 0.56, \qquad m_N^{\Delta^{(3)}} = \frac{2}{2^2}(N_{14}^1) = 0.38. \tag{S8}$$

The corresponding motion vector will then either be

$$\boldsymbol{m} = \left[m_R^{\Delta^{(1)}}, m_R^{\Delta^{(2)}}, m_R^{\Delta^{(3)}}\right], \tag{S9}$$

or

$$\boldsymbol{m} = \left[m_N^{\Delta^{(1)}}, m_N^{\Delta^{(2)}}, m_N^{\Delta^{(3)}}\right]. \tag{S10}$$

References

[1] Z. Wang, A. C. Bovik, H. R. Sheikh, and E. P. Simoncelli, "Image quality assessment: From error visibility to structural similarity," *IEEE Transactions on Image Processing*, vol. 13, no. 4, pp. 600–612, 2004, doi: 10.1109/TIP.2003.819861.

[2] S. van der Walt *et al.*, "scikit-image: image processing in Python," *PeerJ*, vol. 2, p. e453, 2014, doi: 10.7717/peerj.453.

[3] E. Ilg, N. Mayer, T. Saikia, M. Keuper, A. Dosovitskiy, and T. Brox, "FlowNet 2.0: Evolution of Optical Flow Estimation with Deep Networks," in *2017 IEEE Conference on Computer Vision and Pattern Recognition (CVPR)*, 2017, pp. 1647–1655. doi: 10.1109/CVPR.2017.179.
